# A Case of Progressive Muscular Atrophy from Lead-Poisoning

**Published:** 1867-05

**Authors:** 


### SELECTIONS.

### *A Case of Progressive Muscular Atrophy from Lead-Poisoning.* Under the care of Dr. Wilks.

The patient whose case is here related, is still an inmate of the hospital. Her improvement, however, has been so steady and marked for some time past that there can be no object in delaying the publication of her case, which is one of peculiar interest. It chanced that we saw her whilst she was being admitted into the hospital, and the change which is now to be observed in her appearance is indeed remarkable. Our account of the case is partly derived from notes taken by Mr. Branford Edwards, Dr. Wilks's clinical clerk, and partly from our own observation.

Amelia C——, aged twenty-four, single, admitted on the 4th of July, 1866, in a state of extreme emaciation. She lies on her back, perfectly helpless, and presenting literally the appearance of a skeleton. Every muscle in the body is wasted to a very unusual and remarkable extent. Those of the back share in the general atrophy, which, however, is perhaps most of all marked in the muscles of the hands and arms. The fingers are flexed, giving the characteristic "griffin's claw" appearance, the flexion phalangeal and not the metacarpophalangeal joints. The interossei seem to have entirely disappeared so that the finger and thumb of an observer can be made between the metacarpal bones. The radius and ulna can be made out throughout their length quite as distinctly as though covered only by integument. The legs and feet are in a very similar condition. So wasted are the abdominal muscles that the spine can be distinctly felt throughout the lumbar region.

Her history is mainly derived from herself, and is not so clear as might be wished. It seems, however, that she began to menstruate at fourteen, and at that time was particularly fat. She lived at home with her parents, assisting in the household work, and although not very strong, enjoying very fair health. About five years ago she would appear to have sustained a great mental shock from the death of her mother. She went on a visit to London, and there suffered from what she calls "inflammation of the bowels," which laid her up for two months. She has never been so well, she thinks, since that attack; but she was able to get about and work as usual. Her condition was one of debility, with irregular menstruation and slight histeria. In November, 1864, she entirely ceased menstruating. In February, 1865, she left her home, owing to her father marrying again, giving up his farm, and taking a situation. She seems to have fretted very much at these changes and at her failing health. For nights together, she says, she did not sleep, thinking of her troubles. She got weaker, and lost her appetite. At this time (seventeen months before admission) she went to stay with a sister living at a village in Kent. Soon after her arrival (we learn from the doctor who attended her) she suffered more than once from attacks of histeria; and she had not been there more than six or seven weeks when her health became worse than ever. She suffered from constant vomiting, abdominal pain, and gradual increasing weakness in her extremities, which she first noticed, she thinks, in her hands and arms. She lost all power over her limbs, and lay helpless in bed. In July, 1865, she lost her voice for about eight weeks, at the end of which time it returned. From this time she never left her bed for a year. At first there was great constipation of the bowels, sometimes no action taking place for a month. This was succeeded after some months by diarrhœa, and she passed her motious involuntarily. She never, however, lost control over her bladder. In this helpless, emaciated condition she lay, vomiting her food sometimes for weeks together, until her admission into Guy's Hospital in July, 1866. It must be noted, however, that her condition at that time was not quite so bad as it had been in the January preceding. There was, if anything, a little more power over the limbs.

On examination, Dr. Wilks discovered a blue line along the margin of the lower gum, and a less distinct one on the upper. On making inquiry into the possibility of exposure to lead, it was found that the water which she had been drinking came from a well close to the house, and was pumped up through a leaden pipe. It was surface water, and was very soft. A quantity was sent up for analysis, and Dr. Stevenson found that it contained a very minute amount of lead. It was ascertained that she had been living in one of two semi-detached cottages, each of which was supplied from the same well, but owing to its position, there was twice the length of lead piping conducting the watre into the adjacent house as into that which she occupied. The former was inhabited by a gardener and his wife, who had never suffered any ill effects. The latter contained, besides the patient, five persons: her sister and brother-in-law with three children. The only thing that could be learned about them was a doubtful history of weakness in one wrist of which the sister was understood to complain.

The patient was ordered to take five grains of iodide of potassium three times a day, and to be galvonized (faradisation) daily. For a month after admission she suffered much from looseness of the bowels and vomiting. The diarrhœa was apparently due to muscular relaxation. Under strong induced currents the flexor muscles were found to contract but there was no action in the extensors. By slow degrees the electro-motility of the muscles improved. On the 6th of September it is noted that "she is going on well; the muscles are obviously increaing in size. In October she was able to be moved into another ward. She could use the hands a little but could not stand. She became able to write and employ knife and fork. In November she walked across the ward with the aid of a chair. On the 3d of December she menstruated for the first time during two years. Rapid improvement took place during the month; and when we saw her a few days since she was able to walk about without assistance, and her hands presented an appearance which was nearly natural. She is still very thin, but she has lost that look of frightful emaciation which formerly belonged to her.

Dr. Wilks believes the case to be one of muscular atrophy from lead. insusceptibility of the extensor muscles to the induced current strongly favors this view, which is confirmed by the finding of lead in the water consumed, and the presence of the blue line on the gums.—*London Lancet.*

